# Indigenous pedagogies and online learning environments: a massive open online course case study

**DOI:** 10.1177/11771801221089685

**Published:** 2022-04-06

**Authors:** Danielle Tessaro, Jean-Paul Restoule

**Affiliations:** Department of Indigenous Education, University of Victoria, Canada

**Keywords:** inclusive online education, Indigenous education, Indigenous pedagogies, Medicine Wheel, MOOCs, online learning

## Abstract

This study is based on a massive open online course titled *Aboriginal Worldviews in Education*, which was created and instructed using various Indigenous pedagogies. Despite significant pedagogical differences between massive open online course and Indigenous pedagogical learning environments, this study builds the case that various Indigenous pedagogies *can* effectively be incorporated in a massive open online course. The study found that holistic pedagogies were effectively applied by centering course creation and instruction around Medicine Wheel teachings. The article details the various experiential and self-reflective activities that were applied to the massive open online course, and that were found to effectively address the spiritual, emotional, and physical quadrants of the Medicine Wheel that are normally overlooked in courses that stress intellectual learning. The article also suggests directions for the development and redevelopment of massive open online courses to better include Indigenous pedagogies.

## Introduction

The following case study draws on literature discussing Indigenous pedagogies (Castellano, 2000; Couture, 1991; Grande, 2015; Restoule & Chaw-win-is, 2017; L. B. Simpson, 2011; L. R. Simpson, 2000; G. H. Smith, 2003), to depict how certain Indigenous pedagogies were applied to the creation and instruction of a massive open online course (MOOC). The article also draws on original survey and in-course data from MOOC users, to discuss how the applied Indigenous pedagogies were experienced by learners. The MOOC that forms the basis of this project is titled *Aboriginal Worldviews in Education*, offered on Coursera since 2013. In its initial offering in 2013, this MOOC drew in over 23,000 registered learners, and was led by Dr Restoule of the University of Toronto. The initial offering saw relatively high completion rates of 13%, versus the average completion rate of 4% at the time (Jordan, 2015). Following the initial offering, the course has remained open first in an archived form, and then from 2017 in an on-demand mode, meaning that the course can be taken by new registrants, albeit with no active instructor. Thousands of learners per year have registered in *Aboriginal Worldviews in Education* in the on-demand format. It should be noted that the high interactivity, possible in the initial offering, was not repeated because of inadequate institutional supports. No course release or assistants were offered by the host institution to run the course a second time.

Although the numbers sound high when compared to most forms of learning, thousands and tens of thousands of registered learners is typical for MOOCs. Across the three leading MOOC providers Coursera, edX, and FutureLearn, there are well over 100,000,000 registered users, with that number steeply increasing since the start of the COVID-19 pandemic (Shah, 2020). MOOCs represent a unique and relatively new form of learning, entering into prominence around 2011 and 2012, the latter year referred to as “the year of the MOOC” (Pappano, 2012, para. 1) by the *New York Times*. The uniqueness of MOOCs stems from the fact that they are led by University and College Professors, but are free and accessible to anyone with an internet connection (Sharrock, 2015). Due to their high enrollments and mass accessibility, when it comes to pedagogical considerations, MOOCs are in a category of their own. MOOC instructors and course creators must be considerate of their uptake by diverse audiences, taking the course with a range of educational backgrounds, interests, and skill sets. For MOOC creators and instructors, learner contexts are essentially unknown.

Indigenous pedagogies have been called upon by scholars, such as G. H. Smith (2003) as a necessary contribution to bridging university learning with Indigenous communities, such as by “creating indigenous space in the academy for indigenous development and advancement” (A Call to Theory section, para. 14) and “developing curriculum options that are built around indigenous interests first and foremost” (A Call to Theory section, para. 19). This study acknowledges the diversity of Indigenous pedagogies, originating from a diverse range of Indigenous knowledges, cultures, contexts, and localities (Dei et al., 2000; Restoule & Chaw-win-is, 2017). And while there is no singular or prescribed set of Indigenous pedagogies, literature on the subject tends to emphasize certain characteristics. One prominent characteristic is the emphasis on teaching and learning as immersed in community, place, and context (Cajete, 1994; Hall, 1976; Restoule & Chaw-win-is, 2017).

Within high-context learning environments, Indigenous pedagogies also underline a high level of familiarity between teacher and learner(s), where the teacher tailors their teaching practice according to learner needs and existent knowledge levels (Styres & Zinga, 2013). Therefore, the extremely low-context nature of MOOC learning environments (McLoughlin & Oliver, 2000) presents a challenge for Indigenous pedagogical approaches. With this difference in high-context learning environments for Indigenous pedagogies, versus extremely low-context learning environments for MOOCs, proponents of Indigenous education are likely to doubt the compatibility of Indigenous pedagogies and MOOCs.

Additional Indigenous pedagogical characteristics that present challenges for incorporation into online learning environments include land-based teaching and learning, as well as experiential education (Absolon, 2011; Cajete, 1994; Castellano, 2000; L. R. Simpson, 2000). Yet, as more and more higher education courses shift to fully or partially online options, studies that examine Indigenous pedagogies in online learning environments have become crucial for positioning Indigenous “ways of knowing as being relevant and significant in the ‘elite’ knowledge production and reproduction ‘factories’” (G. H. Smith, 2003, The Need to Centralize section, para. 6). For McLoughlin and Oliver (2000), “the design of Web based instruction is not culturally neutral, but instead is based on the particular epistemologies, learning theories and goal orientations of the designers themselves” (p. 58). Thus, as MOOCs continue to grow in scope (Baturay, 2015; Shah, 2020), it is important that studies consider the inclusion of culturally diverse pedagogies, including Indigenous pedagogies. This is especially critical for MOOCs, as they have been criticized for their lack of attention to non-Western dominated, non-Eurocentric pedagogies, and curricula (Gais, 2014).

More and more university-level courses are being offered online, a trend that saw a steep increase during the COVID-19 pandemic. Many educators interested in, or used to teaching with, Indigenous pedagogies may be tempted to (re)abandon these pedagogies in fear that they will conflict with the online environment. For instance, the Associate Dean Experiential Education at the author’s institution resigned when online courses were mandated due to the pandemic in 2020, out of the firm belief that experiential pedagogies could not be brought to online teaching. Experiential-based learning is a component of Indigenous pedagogies. Yet, to re-negate Indigenous pedagogies in the face of online learning, instead of attempting to bridge online learning and Indigenous pedagogies as in this study, would be to substantially limit “efforts to disrupt the settler foundations of both school and society” (Grande, 2015, p. xv). The following case study will build the argument that forms of Indigenous pedagogical practice *are* possible in the online environment, even in one so diverse as the MOOC environment. The example we use is an MOOC about Indigenous education and the article begins from an assumption that many educators might be skeptical about the ability to teach in an Indigenous way online or in virtual environments. For this reason, we choose to talk about an example where it might seem inherently paradoxical that Indigenous education can be translated into online learning. Our concern in the article is with seeing how Indigenous pedagogies might be applied in online course design but we have not tried it with a course that has no Indigenous content. Bringing Indigenous pedagogies to teaching online courses that are not Indigenous may raise a number of additional questions and concerns often asked when non-Indigenous teachers want to use Indigenous pedagogies in the classroom (Carroll et al., 2020). They are not the focus of the present article.

Our work in this article is to depict the ways in which Indigenous pedagogies were effectively brought to the creation and instruction of *Aboriginal Worldviews in Education*. Findings as to the “effectiveness” of the pedagogies emerge from the data that are derived from registered users. Our determination of effectivity of pedagogies is based on whether feedback from learners demonstrate a mirroring of intent—for instance, when an attempt to engage emotions is reported back by learners saying the activity produced an emotional learning, the design had the intended result. When the design of activities and materials is meant to provoke transformative learning and learners report that they have been *transformed* or moved to take action for social justice, then we deem the pedagogical design to have been effective. The pedagogies that are cross-referenced in the findings section of this article, which will speak to the pedagogical decisions for the MOOC, primarily draw from Anishinaabe-centered approaches (Absolon, 2011; L. B. Simpson, 2011, L. R. Simpson, 1999, 2000). This is due to the culture of the MOOC course designer and instructor, Dr Restoule. Specifically, the article will depict how Indigenous, holistic pedagogies (Bopp et al., 1989; Castellano, 2000; Restoule & Chaw-win-is, 2017) informed by the Medicine wheel (Battiste & Barman, 1995; Bell, 2014; Bopp et al., 1989; Nabigon & Mawhiney, 1996; Toulouse, 2016), experiential pedagogies (Cajete, 1994, 1999; Castellano, 2000; L. B. Simpson, 2011; L. R. Simpson, 2000) self-reflection or *turning inward* (Absolon, 2011; Castellano, 2000) and screenside chats or informal instructor videos demonstrating relationality and interactivity, were all effectively applied to *Aboriginal Worldviews in Education*. Based on these findings, we will then discuss opportunities for the development and redevelopment of MOOCs to effectively incorporate elements of Indigenous pedagogies. This part of the article aims to be of use to educators aspiring to bring Indigenous pedagogies online.

A review of the literature reveals a small range of studies on MOOC pedagogy (Abeer & Miri, 2014; Guo et al., 2014; Hone & El Said, 2016; Liyanagunawardena et al., 2013; Najafi et al., 2015, 2017), as well as a small body of literature on Indigenous online education (Fredericks et al., 2015; Kutay et al., 2012; McLoughlin & Oliver, 2000; Pulla, 2017; Reedy, 2019; Tessaro et al., 2018; Wemigwans, 2018). However, there are currently no studies depicting the relationship of Indigenous pedagogies and MOOCs, or even Indigenous education more broadly in conjunction with MOOCs or other large online course formats. Our study will contribute to addressing this gap in the literature, and may be useful to instructors of online courses, MOOC instructors or designers, academic programs, or those interested in Indigenous education, during an era of an unprecedented rise in the number of courses taught online.

## Methods

The study is grounded in an Indigenist research paradigm (Wilson, 2007, 2008), and is informed by critical pedagogical approaches to knowledge production, developed by scholars in Indigenous education and research methods (Absolon, 2011; Kitchen & Raynor, 2013; Kovach, 2009; Restoule, 2011; L. T. Smith, 2012; Styres & Zinga, 2013). The Indigenist paradigm framed this research through the various principles described by Wilson (2007), such as “theories developed or proposed must be grounded in an Indigenous epistemology,” and “methods used will be process-oriented” (p. 195). Wilson also indicates that *transformation* should be a research outcome, and indeed *transformation* is discussed as a research outcome that took place through the MOOC. The guidance of Indigenous scholars and elders is another principle of the paradigm described by Wilson and utilized in this project, especially in relation to the Medicine Wheel teachings.

This project was conducted as a qualitative case study of the *Aboriginal Worldviews in Education* MOOC, allowing for a rich, in-depth assessment of Indigenous pedagogies for MOOC course creation and instruction. According to authors, such as Hone and El Said (2016) and Raffaghelli et al. (2015), case study approaches are common in studies examining MOOCs. Data for this project concerning course creation were derived from members of the *Aboriginal Worldviews in Education* team from the Ontario Institute for Studies in Education, at the University of Toronto. Information obtained from these personnel was derived through various forms of personal communications, such as informal interviews and emails. During these conversations, course creators discussed their course design strategies and rationales. Data from learners was obtained through survey data from the initial offering, discussion forum posts from the initial offering, as well as user reviews of the course provided from Coursera. The user reviews have been provided since the initial offering, and were provided both privately, in the case of course completers prompted to review the course by Coursera, and publicly, in the case of those who posted public course reviews on the platform. Permission to use survey data was obtained from participants when they enrolled in the course in 2013. All learner data obtained for the purposes of this analysis were provided voluntarily, without specific inquiries directed toward the subject of the article: Indigenous pedagogies. That is, learners responded to broad prompts regarding their experiences with the course, as well as specific questions regarding how they felt about a specific assignment or a specific component of the course, for instance. Yet, even without specific prompts inquiring of learner experiences with various Indigenous pedagogies, the analysis found that many learners spoke of pedagogy-specific experiences. The survey data were collected in the initial course offering in 2013 when the instructor was more involved and interactive, which may have affected how learners rated the experience. There were a high number of Indigenous registrants, in the hundreds, but the majority were non-Indigenous, according to demographic information collected as part of registration. For more information on where the learners came from for this MOOC, see Brugha and Restoule (2016).

Analysis of the learner data was conducted using several rounds of document analysis, which sought to assess threads and themes among survey data, discussion forum data and course feedback in regard to the Indigenous pedagogies and course design strategies that had been indicated by the course creation team. Essentially, where Indigenous pedagogies as assessments or activities were indicated by the course design, we searched the learner-derived data in our analysis to cross-reference threads and themes that were pertinent to the named pedagogies. The learner data were also assessed multiple times for Indigenous pedagogical themes that may coalesce into findings in their own right, independent of strategies indicated by the course design team. Through each type of analysis—guided and unguided—findings emerge, and are outlined below.

## Results

### Holistic course design and the Medicine Wheel

Various authors indicate that Indigenous education is meant to engage each learner on a holistic level (Bopp et al., 1989; Castellano, 2000). For Restoule and Chaw-win-is (2017), Indigenous education emphasizes “the importance of seeing the whole before its parts” (p. 14). The Final Report of the Royal Commision on Aboriginal Peoples describes Indigenous pedagogies and learning processes as having “intellectual, spiritual, emotional, and physical dimensions” (1996, as cited in L. R. Simpson, 1999, p. 34). For *Aboriginal Worldviews in Education*, holistic course design was applied through usage of the Medicine Wheel as an organizing principle. The Medicine Wheel as a holistic approach to areas, such as education, social work, or even addictions counseling, has been described by various Indigenous authors (Battiste & Barman, 1995; Bell, 2014; Bopp et al., 1989; Nabigon & Mawhiney, 1996; Toulouse, 2016). Much of the lead MOOC instructor’s understanding of the Medicine Wheel was derived from elder’s in-person teachings, such as at Elder’s lodges, gatherings, and conferences. Elders Dr Restoule learned Medicine Wheel teachings from include Michael Thrasher, Vera Martin, Lillian Pitawanakwat, Jim Dumont, Pauline Shirt, Myra Laramee, and Debby Danard Wilson

The exact format and wording of the Medicine Wheel varies across cultures, and from teaching to teaching, but one constant is the use of four *quadrants* of the wheel, each coming together in the middle, where balancing the components, striving for unity and harmony is a key teaching reminding us how the whole is greater than the sum of its parts. In Dr Restoule’s understanding, the Medicine Wheel tells us we are composed of four aspects of being: *spirit*, or intuition, *emotion*, or feelings, *body*, or physical aspects of being, and *mind*, or intellectual aspects of being. To foster holistic pedagogies in the MOOC, the course was designed with each of those quadrants in mind. Since in this case, the Medicine Wheel was being utilized for a course—and for all courses, the primary objective would broadly be learning—the mind, or intellectual aspects of the Medicine Wheel for course design do not require much explanation. Learners’ minds and intellects were engaged through readings, assessments, and short quizzes based on the material. There were two quizzes in *Aboriginal Worldviews in Education*. They were both multiple choice and could be re-taken up to 100 times. Moving forward, the following paragraphs will describe how the remaining three quadrants of the Medicine Wheel, that of emotion, spirit and body, the ones often underutilized in Western teaching and learning, were used to inform decisions for the MOOC course design.

### Holistic pedagogies: Emotion

An important part of the holistic approach and the use of the Medicine Wheel was to engage learner emotions. In addition to emotionally evoking topics, such as learning about residential schools, engaging learner emotions was incorporated into the MOOC through a *loss* activity. The loss activity was learned from another teacher at OISE who does not remember where they learned it, but it has been used effectively to teach about the erosion of knowledge and genocide. The MOOC designers did not create or originate the activity. The activity takes place in Week 2 of the course, and is not a formal assessment. For the loss activity, the instructor asks students to write down ten names of people close to them. Then, the instructor asks them to cross out nine of the names. Through the activity, learners may imagine what it would have been like to lose 90% of people closest to them, as so many Indigenous peoples did in North America in the first 50 years of European contact.

The learner data showed substantial evidence that the course engaged learner emotions or *whole selves*, in various ways. Examples from the learner data include the following statements:

- “The thing I have loved the most about this course is the emotion that has come along with it. I have laughed, I have cried, but I think the most important thing is I now have a different understanding for the who, what, where, when, and why. And will go away with this wonderful experience in my heart.”
- “I don’t know why, but it seems like people are engaging more emotionally in this course.”
- “I stayed in it because I felt it presented a depth of knowledge that went beyond the cerebral to the internal.”
- “You are offering a lot of healing to me through your videos and words and spirit!”
- “Of course the content goes to one’s heart, but it was more than that.”

While the above provide a sample of the types of comments elicited by elements of the course, one can see student involvement moving beyond the intellect to include emotions and more holistic engagement.

Literature that discusses Indigenous pedagogies as holistic often refers to the *transformative* nature of a holistic learning experience (Cajete, 1994, 1999) Several learners referred directly to the *transformational* nature of their learning experience, which speaks to the objective of engaging learners holistically. For instance:

- “This is my first Coursera experience and it was informative, thought-provoking, and emotionally draining all at once! It was a roller-coaster taking me from heights of excitement at all the new and wonderful things I was learning and lows of shame and frustration at some of the current social and political decisions that are continuing the abusive relationships of the past . . . .Transformative is the best word I can find for the experience.”
- “This course has changed who I am, and how I’ll be . . . .Thanks for making me a better person!”
- “[a]in’t none of us walking out of here quite the same as when we walked in.”
- “This course has changed the way I see everything.”

Indeed, this transformation of personal and political being was a desired outcome of the course for the MOOC designers. It was not enough to have students learn some facts; the intent was to challenge normative assumptions and worldviews.

### Experiential pedagogies and turning inward: spirit and body

Course design also sought to bring the Indigenous pedagogy of experiential learning to the MOOC. Experiential learning is often cited as an Indigenous pedagogy (Cajete, 1994, 1999; Castellano, 2000; Restoule & Chaw-win-is, 2017), including in the literature that speaks directly to Anishinaabe pedagogies (Absolon, 2011; L. B. Simpson, 2011; L. R. Simpson, 2000). For instance:

“Experience continues to be a fundamental principle of Anishinaabe learning processes” (Cajete, 1999, as cited in L. R. Simpson, 2000, Learning by doing section, para. 2), and “The elders focus on learning from one’s experience through respectful and patient observation” (Couture, 1996, as cited in L. R. Simpson, 2000, Learning by doing section para. 4).

Due to the virtual, asynchronous nature of MOOCs, and their high enrollments, certain conventional, hands-on forms of experiential learning are impossible. Instead, experiential learning was embedded into *Aboriginal Worldviews in Education* through assessments that required students to reflect on, envision and/or share certain experiences. Castellano (2000) highlights the Indigenous pedagogical importance and effectiveness of sharing personal experiences with others, for instance, “[o]ne of the most effective ways of learning was to listen to the stories of personal experience that were told” (p. 27). Thus, assessment strategies were designed with experiential pedagogies in mind, to the extent that could be offered online, such as through envisioning and sharing an experience.

For example, one assessment required students to describe a familiar gathering place in their locality, such as a school, workplace, or sports facility, as if they are experiencing it for the first time. The assignment, titled *Making the Familiar Strange*, aimed to have learners break open the authoritative, ethnographic voice that is so often used to describe—and consequently alienate—Indigenous people and cultures. The *Making the Familiar Strange* assessment takes place after students engage with a reading on ethnographic methods, short lectures, and a short film titled *Babakiueria* (Featherstone, 1986). The film depicts a satirical role-reversal in describing the daily activities and plights of White Australians, in the tone so often used by White Australians to describe Aboriginal people. The assessment requires the application of what students have watched and learned to a regular experience of their own: attending a familiar place.

Our study found that this attempt at incorporating the Indigenous pedagogy of experiential learning into an MOOC was effective. For instance, one learner comments that “I get to engage experientially with the material and my fellow learners and gain from their insights and interpretations of their experience of the various topics and resources.” Another learner commented, “It was quite astounding actually how visceral the experience was . . . given that it meant connecting to you and the content through a machine.” We believe these statements exemplify the compatibility of MOOC course design with *experiential* aspects of Indigenous pedagogies. Although the *Making the Familiar Strange* activity was not physical or hands-on, experiential learning was engaged through detailing an everyday experience.

The practice of learning by relating teachings to personal experience, or to the *self*, bridges this form of experiential pedagogy to another Indigenous pedagogy of “turning inward” (Castellano, 2000, p. 28), also referred to by Absolon (2011) as the “self-as-central” (p. 57). For instance, according to Ermine (1995, as cited in Castellano, 2000) “those who seek to understand the reality of existence and harmony with the environment by turning inward have a different, incorporeal knowledge paradigm that might be termed Aboriginal epistemology,” (p. 28). For Absolon (2011), “the self-as-central” emphasizes the importance of “understanding the self in relation to the whole. In many cases, the Indigenous searchers utilized a self-referential and experiential approach to gathering knowledge” (p. 57).

The goal of experiencing the familiar-made-strange was to simulate the othering process that happens to Indigenous people, and how canonized knowledge fixates Indigenous experience creating *authentic* identities, which consequently limit Indigenous freedom to develop and grow organically. With identities being fixed, Indigenous people demonstrating change can be equated with losing authenticity, or becoming *less* Indigenous. Experiencing the ethnographic voice, as this assignment aimed to do, opens up the learners’ eyes to how Indigenous identities are fixed and made knowledgeable, which is to exert power over them.

Appreciation and recognition of the turning inward or self-as-central pedagogy was expressed in the learner data. For instance:

- “learners become more and more directive in their own learning and are encouraged to interweave their own life experiences with what they learn”
- “there’s more going on than what is obvious, learning comes from the inside”
- “For the ways you tied it together and made all of us examine our selves and own our [obscenity], I Thank You.”

In another learning activity, students were asked to post a narrative or photographs to the discussion board about a place that was meaningful to them. This activity was meant to blend threads of self-reflective and experiential Indigenous pedagogies. Reflecting on and sharing a meaningful place was also meant to evoke the *spirit* aspect of the Medicine Wheel. The purpose of this pedagogy in the course was to turn inward, to show something about where you are from and why you have connections to that place, before linking this experience to the Indigenous experience of what it means to be connected to a place for thousands of years.

The meaningful place and the “making the familiar strange” activities were also rationalized as the *physical* quadrant of the Medicine Wheel, as they required envisioning or visiting a physical location with your physical body and senses. However, since the creation of *Aboriginal Worldviews in Education* in 2013, the course instructors have implemented online experiential and *physical* pedagogies in other online courses that require learners to go directly to the land, to reflect on place or to find story sites or gather medicines. These activities would be suitable for the redevelopment of this MOOC, or in making future MOOCs. Land-based Indigenous pedagogies are discussed in the later section on opportunities for redevelopment.

### Screenside chats

An unintentional Indigenous pedagogical approach that was found to be effective, was the instructor’s dissemination of *screenside chats* on a weekly basis in the initial run of the course. The instructor posted an unproduced, unedited video at the end of each week, addressing questions that had been posted and upvoted in the discussion forums. The screenside chats were an adaptive component of the course, not initially planned, but added through the course of instruction. They were posted as optional, additional resources, set apart from the pre-filmed, edited short lecture videos of each weekly module. Screenside chats were not part of the initial Medicine Wheel approach to Indigenous pedagogical course design. Yet, they were found to have fostered a sense of community and responsiveness, in an online learning environment that threatens to be largely void of such characteristics.

Themes emerged from the learner data that indicated that learners experienced the screenside chats in ways that were consistent with literature on Indigenous pedagogies. For instance, some learners expressed that they felt the instructor responded to them in a *non-judgmental* way and *respected* their opinions. *Respect* is one of the Five Rs for Indigenous pedagogy (Kirkness & Barnhardt, 2001; Restoule, 2008; Tessaro et al., 2018), the others being responsibility, relevance, reciprocity, and relationships. Based on the learner data, it seemed that the screenside chats fostered a sense of “learning, communicating, and working in relationship, based as it is on equality and mutual reciprocity” (Cajete, 2015). Another learner commented, “despite being so far removed from Canada, I feel that I am sitting in the classroom and being spoken to personally.” Learning that feels personable and interactive can often be lacking in an MOOC environment. Ultimately, the screenside chats were believed to have contributed to a more intimate awareness of the teacher by the learners (Styres & Zinga, 2013; Tessaro et al., 2018). Informal instructor videos, similar to the screenside chats, are thus discussed below as an opportunity for MOOCs going forward.

### Opportunities for the development or redevelopment of online courses to include Indigenous pedagogies

As a follow up to the key findings outlined above, this section will present suggestions for the development or redevelopment of online courses using Indigenous pedagogies. Guided by the study’s findings and literature on Indigenous pedagogies, this section strives to inspire and provide ideas for educators interested in applying Indigenous pedagogies to MOOCs, or other online learning environments.

### Holistic pedagogies

An appropriate and meaningful starting point for course creators and instructors interested in Indigenous pedagogies is the consideration of a holistic approach. For *Aboriginal Worldviews in Education*, a holistic pedagogical approach was effectively implemented through the Medicine Wheel teachings of engaging learners’ spirit, mind, body, and emotion. Attending to diverse aspects of beings is good pedagogical practice (Bell, 2014). For instance, Graveline (1998, 2000, 2012) discusses the circle as an embodiment of holistic engagement. Overall, the literature on holistic pedagogies and the examples of holistic learning activities described above may provide a useful starting point for MOOC instructors in considering taking up Indigenous pedagogies in a meaningful and culturally relevant way.

### Experiential pedagogies and adding land-based pedagogies

Experiential pedagogies can be difficult to incorporate into an online or MOOC setting. And this article does not suggest that online learning is in any way *better* for experiential pedagogies as compared to in-person learning. Instead, our study aims to show several possibilities for maintaining some form of Indigenous pedagogies rooted in experience (Cajete, 1999; Castellano, 2000; L. R. Simpson, 2000) in an MOOC. Since the creation of *Aboriginal Worldviews in Education*, the researchers have piloted land-based experiential learning opportunities in other online courses, and suggest that these pedagogies would be an improvement for fulfilling experiential pedagogies and the physical quadrant of the Medicine Wheel if they were applied to an MOOC. The suggested improvements build on the type of experiential pedagogy utilized in the case MOOC’s assessments, in that, students still must detail and post an experience; however, they add on by incorporating land-based pedagogical activities.

Land-based learning is an important Indigenous pedagogy that involves connecting to place and community to understand how knowledge, worldview, governance, laws, and ethics emerge from these sources, and has become rooted in Indigenous traditions. For instance, “[t]he plants, the animals, the land, the sky are all potential teachers and careful observation of phenomena and relationships within the whole system is a vital source of knowledge” (Restoule & Chaw-win-is, 2017, p. 12), and, “our worldviews are earth-centered philosophies, express strong ties to the land and hold reverence for Spirit and ancestors” (Absolon, 2011, p. 57). One example of a land-based pedagogy that the researchers have effectively implemented into online courses resulted from adapting to online teaching during the pandemic. Instead of a medicine walk field trip, learners were encouraged to individually go to an outdoor area, find a native plant species, research its history and post photos of the plant and their research findings. This has students connect to the *physical* quadrant of the Medicine Wheel, by moving their bodies in their outdoor environments, and connecting to other physical beings in their environments. It also connects to land-based and experiential pedagogies, and ideally has students engage in their local environments through a perspective that may be new to them. There are many other land-based activities that can be implemented in this vein, by requesting that students engage in their local environments and share findings, reflections or experiences with the group. Thus, adding a *land-based* experiential component is a suggested re-development opportunity for bringing Indigenous pedagogies to an MOOC.

### Informal instructor videos: screenside chats or self-location videos

For Castellano (2000), the *personal* character of Indigenous knowledge entails that learners understand where knowledge is coming from, and who is conveying it. This enables learners to trust the knowledge source, including the source’s ability to share Indigenous knowledge in a safe, appropriate way. As described in the section on *screenside chats*, weekly informal videos posted by the instructor in response to questions from students resulted in some learners feeling more connected to their instructor. Perhaps, the screenside chats enabled some sense of learner familiarity and comfort with their instructor, speaking to Castellano’s belief in the *personal* character of Indigenous teaching and learning. Also, the chats, as well as active forum posting, felt more responsive and relational for the learners than a standard MOOC design of *wind-it-up-and-go*, where the instructor is absent after initial creation.

Adding to the idea of the screenside chats as an Indigenous pedagogy, this article suggests that MOOCs may be developed or redeveloped using similar, informal videos by the instructor. If not the same adaptive, responsive weekly format as the screenside chats, even creating an introductory video by the instructor(s) may fulfill aspects of this pedagogical approach. That is, instructors may share a video describing their background and experience with the learners. This video may effectively replace an “About Us” paragraph, providing a more personable option. The video would support the Indigenous research methodology *self-location* (Absolon, 2011; Kovach, 2009; Kovach et al., 2013), and is an opportunity to support various Indigenous pedagogies in the MOOC environment, including emphasis on relationships and community (Reedy, 2019; Restoule, 2008, 2019; L. B. Simpson, 2011; Tessaro et al., 2018). This suggestion is supported by Reedy (2019), who found “a strong level of teacher presence that is supportive, and culturally and pedagogically appropriate” facilitates Indigenous learning styles in online learning environments (p. 144). Adding elements of teacher presence and personability, such as through screenside chats or instructor self-location videos, is thus a consideration for the development and redevelopment of MOOCs, where instructor presence and learning contexts can be extremely low (McLoughlin & Oliver, 2000).

## Discussion

After uncovering the findings of this study and arguing that Indigenous pedagogies *are* possible in an MOOC environment, it must be acknowledged that for many Indigenous pedagogies, online learning and learning through MOOCs will not be able to replace the benefits of in-person learning. For instance, L. R. Simpson (2000) discusses four techniques that Indigenous people use to teach younger people as: “learning by doing, storytelling, dreaming, and ceremonies” (para. 3). While *Aboriginal Worldviews in Education* was able to incorporate *learning by doing* and learning activities involving story-telling, Indigenous pedagogies, such as L. R. Simpson’s (2000) “dreaming” and “ceremonies” are not able to be incorporated (para. 3). Indigenous ceremonies are generally held sacred (L. R. Simpson, 2000; Wemigwans, 2018), and thus not applicable to an extremely low-context MOOC learning environment. Other aspects of online and MOOC environments that prevent them from being completely ideal for Indigenous pedagogies include assessments that require learners to post on public discussion boards, conflicting with the deeply personal nature of Indigenous knowledge. However, our study argues that even though online teaching and MOOCs do not provide *perfect* environments for Indigenous pedagogies, Indigenous pedagogies can be foundational in these spaces.

Even before online education was deemed incommensurate with Indigenous pedagogies and knowledge, concerns were expressed about Indigenous pedagogies being shared in mainstream Westernized formats, such as books. Yet, it was important that Indigenous researchers and authors strove to overcome the potential conflicts of writing about Indigenous cultures and topics, to help share the information that they did not want to see lost. For instance, in justifying Indigenous teaching and learning through books, Castellano (2000) writes, “traditional media for transmitting aboriginal knowledge have become largely unavailable to many aboriginal people, especially the young. The young people no longer have daily access to experiential learning on the land” (p. 32). Taking the lead from the work of many Indigenous scholars and educators then, this study hopes to build the case that the online education environment should be tackled just the same, so that, Indigenous pedagogies are not hereby excluded from present and future online academic endeavors.

Revisiting the literature on MOOCs, several findings in this study for effective Indigenous pedagogies are consistent with findings for effective non-Indigenous MOOC pedagogies. For instance, correlating with our finding that experiential pedagogies were effectively applied through this MOOC, Najafi et al. (2017) argue that “there is potential for fostering active learning in large and diverse learning environments similar to MOOCs” (p. 61). Similarly, work by Hew (2015, as cited in Hone & El Said, 2016) identified active learning as one of “five features that promote student engagement” (p. 158) in MOOCs. Other features to promote student engagement in MOOCs included “peer interaction,” aligning with some of the assessment activities in *Aboriginal Worldviews in Education* that required learners to post on discussion forums or to peer review other learners’ submissions. Another feature indicated by Hew (2015) was “instructor accessibility and passion” (as cited in Hone & El Said, 2016, p. 158), perhaps speaking to our finding of the effectiveness of the screenside chats. The informal video format of the screenside chats that were suggested as a tool for redevelopment in our study is supported by Guo et al. (2014), whose study of MOOC user engagement found that as compared to formal lecture videos, “informal talking-head videos are more engaging” (p. 41). Similarly, instructor presence and support were also indicated by Abeer and Miri (2014) as “criteria that make MOOCs a constructive learning environment” (p. 318).

## Limitations and directions for future research

This study argued that Indigenous pedagogies can be successfully included in an MOOC environment. However, a limitation to the study is its case study format. This study is based on a course where Indigenous pedagogies were kept in mind throughout the creation of the course, and where the course content is suited to the implementation of said pedagogies. It is conceivable that learners willing to register and engage in a course like *Aboriginal Worldviews in Education*, participate in the discussion forums and provide the feedback were more likely to be open to the pedagogical decisions discussed in this article. Furthermore, as a course that fits broadly into the fields of education and social sciences, written assessments are quite standard, allowing for the assessments described in this article, such as having learners envision and share an experience. Such assessment strategies and pedagogies may be more difficult to transfer to a hard science or math course. This formulates a direction for future research, in that there is an existent gap in the literature to confirm our findings, regarding a wider range of MOOCs. This future research would also help to address another limitation of our study, in that *Aboriginal Worldviews in Education* was first offered in 2013. Creating and studying newer MOOCs for their ability to apply Indigenous pedagogies would help to elucidate the long-term significance and scope of the findings from this case MOOC. Yet, we believe that this study can serve as a useful starting or reference point.

## Conclusion

Despite immense differences in the learning environments that work best for Indigenous pedagogies and MOOCs, this article argues that it is possible to effectively incorporate Indigenous pedagogies into MOOCs. The study followed the implementation of various Indigenous pedagogies into the creation and instruction of the MOOC titled *Aboriginal Worldviews in Education*. For the course, Indigenous pedagogies strived to be holistic (Bopp et al., 1989; Castellano, 2000; Restoule & Chaw-win-is, 2017) and were organized by Medicine Wheel understandings (Battiste & Barman, 1995; Bell, 2014; Bopp et al., 1989; Nabigon & Mawhiney, 1996; Toulouse, 2016). In this context, centering on the Medicine Wheel meant the course should address: spirit, emotion, body, and mind.

The study found that the assessments and course activities, such as the loss activity and making the familiar strange peer-reviewed assignment, were able to address learners’ spirit, emotion, and physical aspects of being through describing a visceral experience. Incorporating activities that required learners to envision and share experiences helped to bring in elements of other important Indigenous pedagogies: experiential learning and self-reflection, or, *turning inward* (Absolon, 2011; Castellano, 2000). Our study also found that MOOC learners experienced the informal videos posted by the instructor, the screenside chats, in ways that are reflected in the literature on Indigenous pedagogies (Cajete, 1999; Kirkness & Barnhardt, 2001; Restoule, 2008; Restoule & Chaw-win-is, 2017; Styres & Zinga, 2013; Tessaro et al., 2018).

Based on what this study found to be effective and where improvements could be made, this article then discussed opportunities for the development or redevelopment of MOOCs to include Indigenous pedagogies. Briefly, the strategies are: (1) course creation using holistic pedagogies, such as through the Medicine Wheel, (2) bringing in experiential or even land-based pedagogies, and (3) informal videos by the instructor, such as screenside chats or self-location videos. The opportunity for land-based pedagogies to be implemented into an MOOC and studied, highlights the importance of continued pedagogical experimentation and research regarding MOOCs, especially as a tool for learning about the uptake of Indigenous pedagogies online. To conclude with the sentiments expressed by Najafi et al. (2017), “[f]irst, MOOCs can be a sand box for testing innovative pedagogical practice (Fischer & Wolf, 2015) to further inform teaching and learning in on-campus courses” (p. 61). Indigenous pedagogies should not be dismissed for their preconceived incapability in online environments. Instead, MOOCs can be used as a *sand box* for these pedagogies, so that, they will flourish in new landscapes.
